# Virtual and augmented reality in critical care medicine: the patient’s, clinician’s, and researcher’s perspective

**DOI:** 10.1186/s13054-022-04202-x

**Published:** 2022-10-25

**Authors:** Raphael Romano Bruno, Georg Wolff, Bernhard Wernly, Maryna Masyuk, Kerstin Piayda, Susannah Leaver, Ralf Erkens, Daniel Oehler, Shazia Afzal, Houtan Heidari, Malte Kelm, Christian Jung

**Affiliations:** 1 Division of Cardiology, Pulmonology, and Vascular Medicine, Medical Faculty, University Hospital Düsseldorf, Heinrich-Heine-University Düsseldorf, 40225 Düsseldorf, Germany; 2 Department of Internal Medicine, General Hospital Oberndorf, Teaching Hospital of the Paracelsus Medical University Salzburg, Paracelsusstraße 37, 5110 Oberndorf, Salzburg Austria; 3 Center for Public Health and Healthcare Research, Paracelsus Medical University Salzburg, 5020 Salzburg, Austria; 4 Department of Cardiology and Angiology, Universitätsklinikum Gießen und Marburg, 35391 Giessen, Germany; 5 General Intensive Care, St George’s University Hospitals NHS Foundation Trust, London, UK; 6 CARID, Cardiovascular Research Institute Duesseldorf, 40225 Düsseldorf, Germany

**Keywords:** Augmented reality, Virtual reality, Critical care medicine

## Abstract

**Abstract:**

Virtual reality (VR) and augmented reality (AR) are aspiring, new technologies with increasing use in critical care medicine. While VR fully immerses the user into a virtual three-dimensional space, AR adds overlaid virtual elements into a real-world environment. VR and AR offer great potential to improve critical care medicine for patients, relatives and health care providers. VR may help to ameliorate anxiety, stress, fear, and pain for the patient. It may assist patients in mobilisation and rehabilitation and can improve communication between all those involved in the patient’s care. AR can be an effective tool to support continuous education of intensive care medicine providers, and may complement traditional learning methods to acquire key practical competences such as central venous line placement, cardiopulmonary resuscitation, extracorporeal membrane oxygenation device management or endotracheal intubation. Currently, technical, human, and ethical challenges remain. The adaptation and integration of VR/AR modalities into useful clinical applications that can be used routinely on the ICU is challenging. Users may experience unwanted side effects (so-called “cybersickness”) during VR/AR sessions, which may limit its applicability. Furthermore, critically ill patients are one of the most vulnerable patient groups and warrant special ethical considerations if new technologies are to be introduced into their daily care. To date, most studies involving AR/VR in critical care medicine provide only a low level of evidence due to their research design. Here we summarise background information, current developments, and key considerations that should be taken into account for future scientific investigations in this field.

**Graphical abstract:**

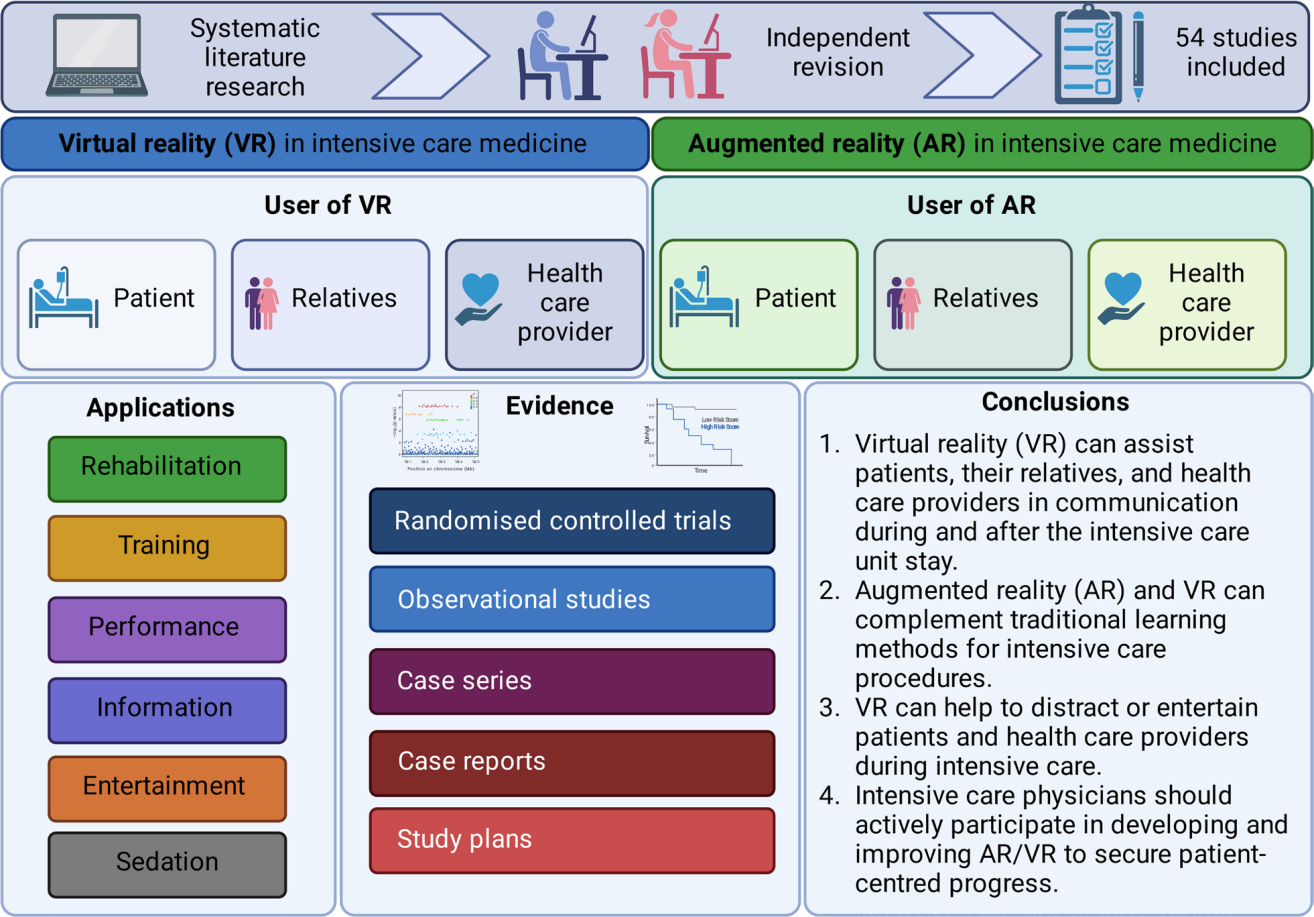

## Background

**Fig. 1:**
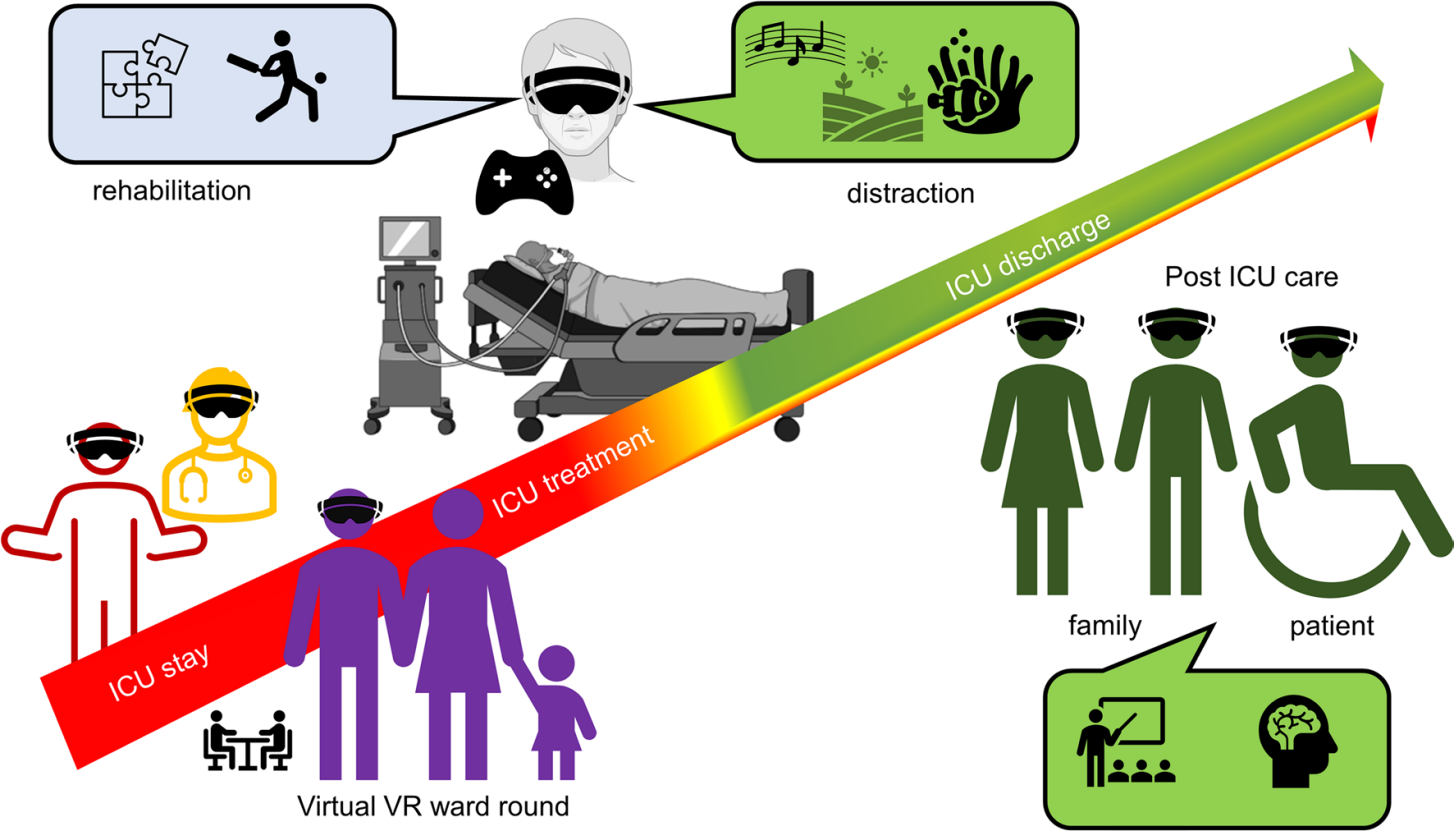
Overview about different users, applications, and the time-course of VR in critical care medicine

**Fig. 2:**
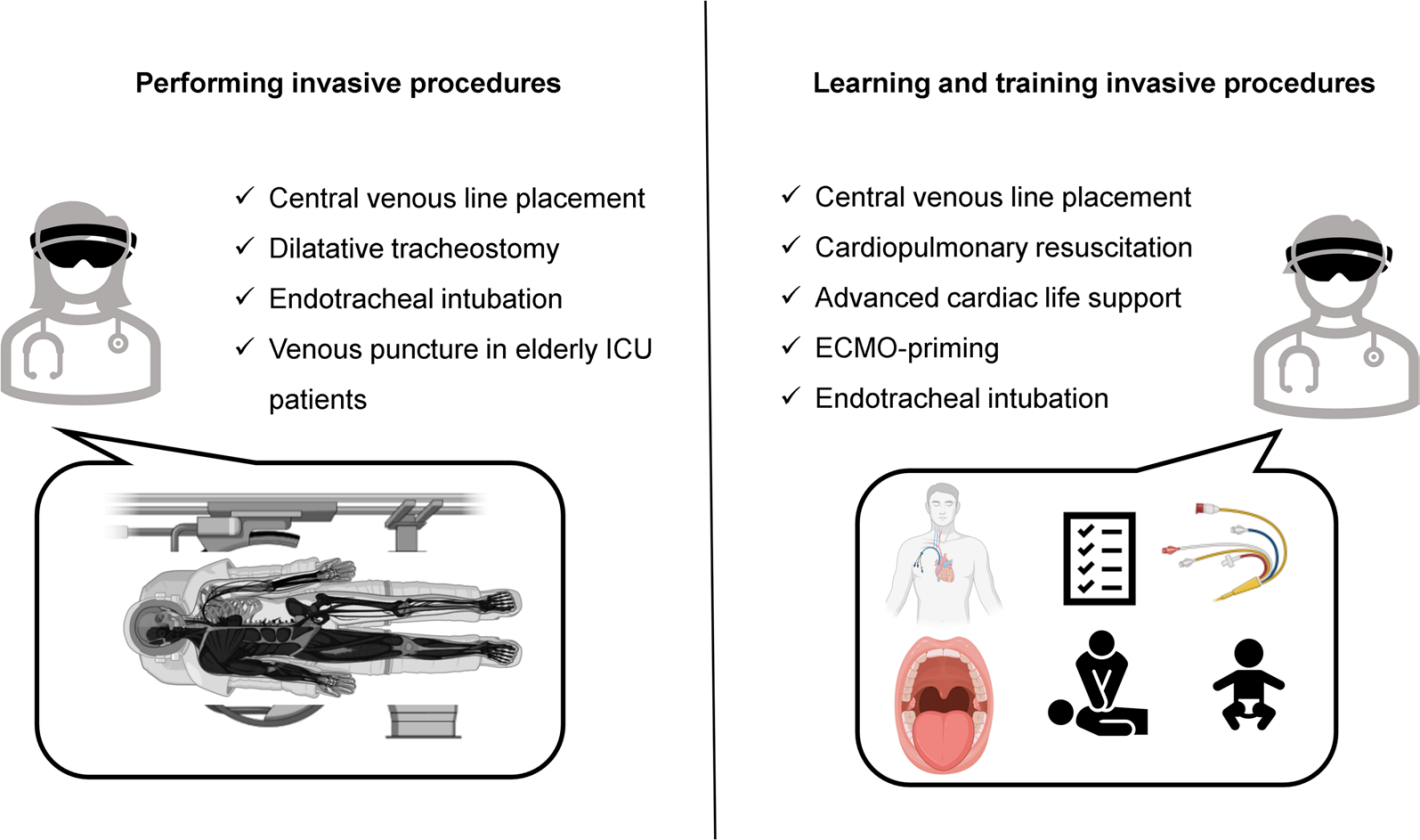
Existing studies where VR/AR applications were used for performing procedures (left panel) and training procedures (right panel)

Both, virtual reality (VR) and augmented reality (AR) are technological breakthroughs which facilitate entertainment and communication worldwide [1]. VR immerses its user completely into a three-dimensional, virtual world, while AR maintains the connection to the “real world” and fuses virtual elements with reality [2]. VR/AR applications have also gained momentum in critical care medicine. Only recently, Critical Care published E-CHOISIR (Electronic-CHOIce of a System for Intensive care Relaxation), the first cross-over randomised controlled trial that clearly shows the benefits of VR on stress, discomfort, and pain in critically ill patients [3]. In addition, VR may help providers learn and improve their practical skills in a protected setting [4], whilst AR offers procedural assistance and continuous surveillance during daily ICU procedures. From a patient’s perspective, VR can alleviate stress, pain [5], and anxiety [6] during critical care, and may also promote coordination, mobilisation, physical, and mental rehabilitation. VR has the potential to improve communication between all stakeholder, including relatives, and thus enable coordinated care and understanding. There are numerous potential opportunities for digital VR/AR applications in critical care medicine (see Figs. 1 and 2, Table 1). However, current VR/AR applications have several drawbacks that need refinement. To date there is limited evidence of benefit in this new emerging field of research.

**Table 1:** Overview of the possible applications of VR and VR for patients, their relatives or health care providers

User	Aim	Description	
		*Virtual reality*	*Augmented reality*
Patient	Distraction/entertainment	VR to distract patients during critical care therapy or during invasive procedures. (+)	(−)
	Treatment	VR to enhance the rehabilitation of patients during critical care therapy. (+)	(−)
	Information	VR to inform the patient during and after the intensive care treatment about critical care therapy. (+)	(−)
Relatives	Education	VR to inform the family or relatives during and after the intensive care treatment about critical care therapy. (+)	(−)
	Communication	VR to establish virtual ward rounds. (+)	AR to help during virtual ward rounds. (−)
Health care provider	Education	For critical care beginners to learn complex critical care subjects. (+)	For critical care beginners to learn complex intensive care subjects. (+)
	Communication	VR to establish virtual ward rounds. (+)	AR to help during virtual ward rounds.
	Stress	VR to help relaxing. (+)	(−)
	Treatment	(−)	AR to help health care providers performing complex intensive care procedures. (+)

( +) = studies available, ( −) = no studies available or approach has currently not been investigated

## Main text

### Virtual reality from the patient’s perspective

#### Alleviating stress and anxiety

**Fig. 3:**
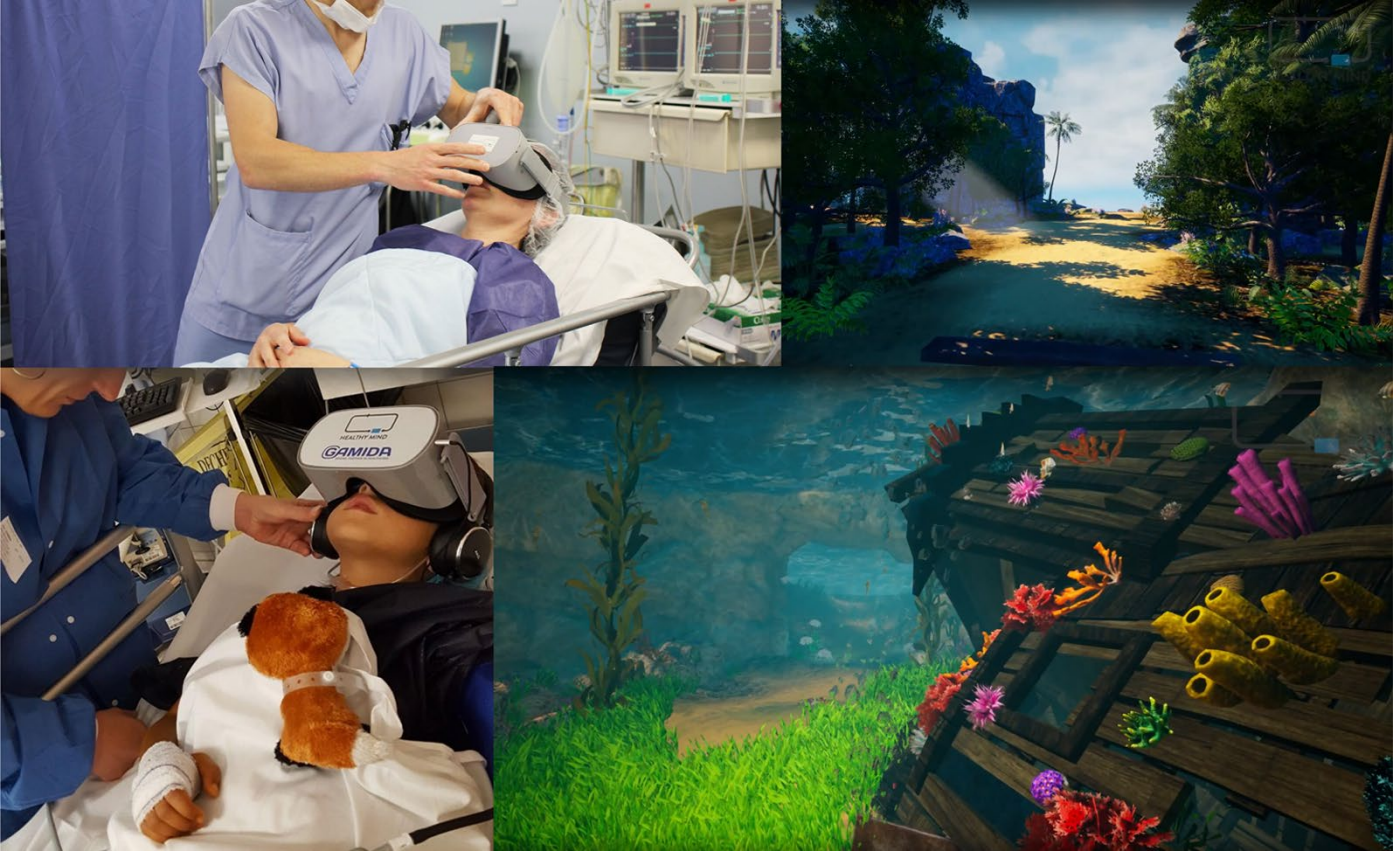
VR with hypnosis used to calm patients during their ICU stay. With permission of Healthy Mind®, France

Patients often experience the ICU as a “hostile” environment due to a number of factors including: excessive noise, loss of self-autonomy and a lack of information [5]. This is augmented by stress and anxiety, both of which are considered to be significant risk factors for the development of delirium. Delirium occurs in 35% to 80% of non-ventilated/ventilated ICU patients and is associated with an increased length of stay and mortality [7]. Since pharmacological interventions often have unwanted, and severe side effects, non-pharmacologic options are of utmost importance to treat, and potentially prevent delirium [8]. ICU stress can be reduced significantly by a calm environment and relaxation techniques. This is an area where VR has been tested. (Fig. 3).

Rousseaux et al. randomised 100 cardiac surgery patients into four arms (control, hypnosis, VR, and VR combined with hypnosis). Every patient underwent one of the techniques for 20 min the day before and the day after surgery. [9, 10]. However, there were no significant differences in the pre-defined outcome measures (anxiety, pain, fatigue, relaxation, physiological parameters, and opioid use) [11]. Further studies are required to investigate potential beneficial effects, and cost-effectiveness. A relative advantage of VR over hypnosis is that VR does not require additional human resources and does not increase the workload of employed ICU staff. By contrast, the previously mentioned E-CHOISIR (Electronic-CHOIce of a System for Intensive care Relaxation) trial found VR to have a positive effect. Sixty alert, and non-delirious ICU patients were randomised into four relaxation sessions (standard relaxation with television/radio, music therapy, and two virtual reality systems with real motion pictures or synthetic motion pictures). There was a significant decrease in overall discomfort and stress response in the synthetic motion pictures group. Both VR systems led to a reduction in anxiety, but only the synthetic motion pictures group reported lower subjective levels of pain. Three incidents (claustrophobia/dyspnoea/agitation) occurred during the VR sessions, but cybersickness was rare [3]. Gerber et al. achieved similar results. The investigators used VR with immersive nature scenes in 33 critically ill patients after cardiac surgery. VR acceptance was high, and most patients reported positive effects on stress. These results were supported by a decrease in respiratory rate during VR sessions [12, 13]. VR has also been found to have a positive effect on sleep quality: in a randomised-controlled trial of 48 ICU patients, VR use resulted in significantly better sleep quality, although the total sleep time and light sleep time did not differ between the groups [14].

In the subgroup of paediatric critically ill patients, VR applications have been shown to have a positive effect on stress, anxiety, and delirium. Badke et al. conducted a cross-sectional, single-arm pilot study with 32 paediatric ICU patients who were provided with simple VR headsets and smartphone videos from a widely available multimedia source for distraction [15]. In this exploratory setting, 82% of parents observed that VR had a calming effect on their child. The same group subsequently recruited 115 critically ill paediatric patients into a comparable study.[16]. During the VR interaction (median duration: 10 min) the majority of patients and their relatives observed a calming effect. However, children returned to their pre-intervention state once the VR application was stopped.

In conclusion, many studies suggest a positive effect of VR on stress, anxiety, and delirium in critically ill patients. To date, the largest, prospective, randomised-controlled trials in this area have shown neutral [11] or positive [3] results.

#### Virtual reality for pain management

**Fig. 4:**
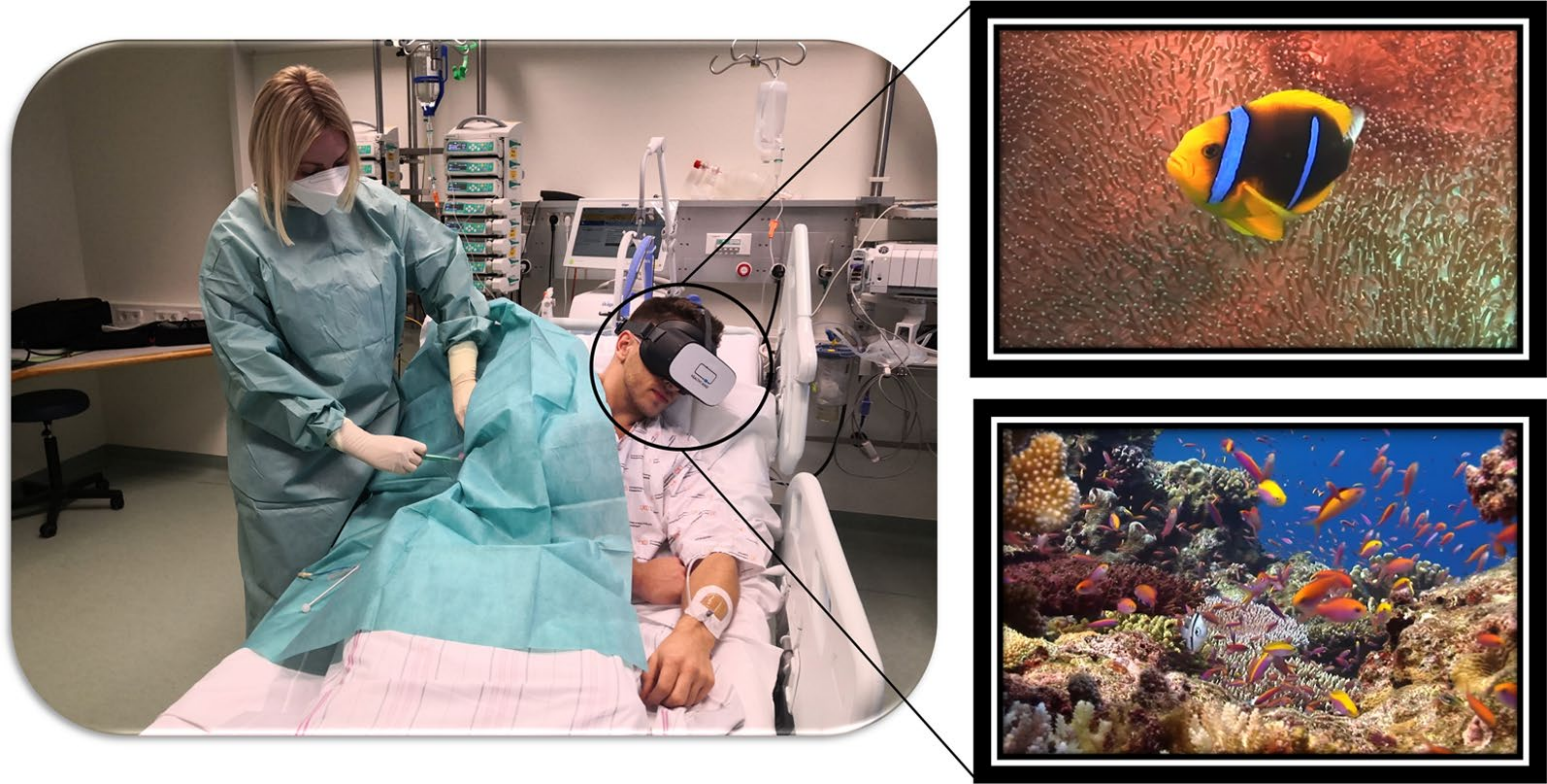
VR for distraction during critical care treatment

Along with anxiety and stress, pain is one of the most common, and burdensome symptoms in critical care patients. The concept of using VR to distract patients during painful procedures emerged in the late nineteen nineties (Fig. 4): There is good evidence for the benefit of VR for the management of chronic [17] and post-operative pain. Mosso-Vázquez et al. enrolled 67 patients after cardiac surgery. Their VR intervention consisted of different immersive environments [18]. After VR sessions, 59 patients (88%) reported a decreased level of pain on a Likert Scale. Furthermore, a systematic review and meta-analysis by Ding et al. including eight randomised-controlled trials [19] found that patients who underwent a VR intervention had lower postoperative pain scores than those receiving standard care. However, there was no significant postoperative pain relief when VR was applied during the pre-operative period. Laghlam et al. evaluated whether VR use in cardiac surgery patients was non-inferior to a combination of nitrous oxide and oxygen. This randomised prospective, non-inferiority, open-label study in 200 patients specifically assessed the degree of pain associated with chest tube removal. VR was inferior to an additionally used inhaled analgetic with regards to the reported level of pain [20]. Hoffmann et al. tested a VR game in 48 burns victims, age between 6 and 17 years old, while their wounds were cleaned. Compared with the control group, the self-reported pain was significantly reduced [21]. However, Faber et al. found that the effect of repeated VR interventions might become less effective after three successive days [22]. According to a study by Hoffman et al. in 11 burn victims, there is a correlation between the “immersive strength” (degree of immersion) of VR and its analgesic effects [23]. Other research groups additionally focused on the feasibility of VR applications in daily clinical practice. Markus et al. required 59 min for VR setup, instruction, therapy, and cleaning [24]. In summary, there is convincing evidence for the positive effects of VR on pain management, especially in burn victims and children.

#### Virtual reality for rehabilitation during the intensive care unit stay

**Fig. 5:**
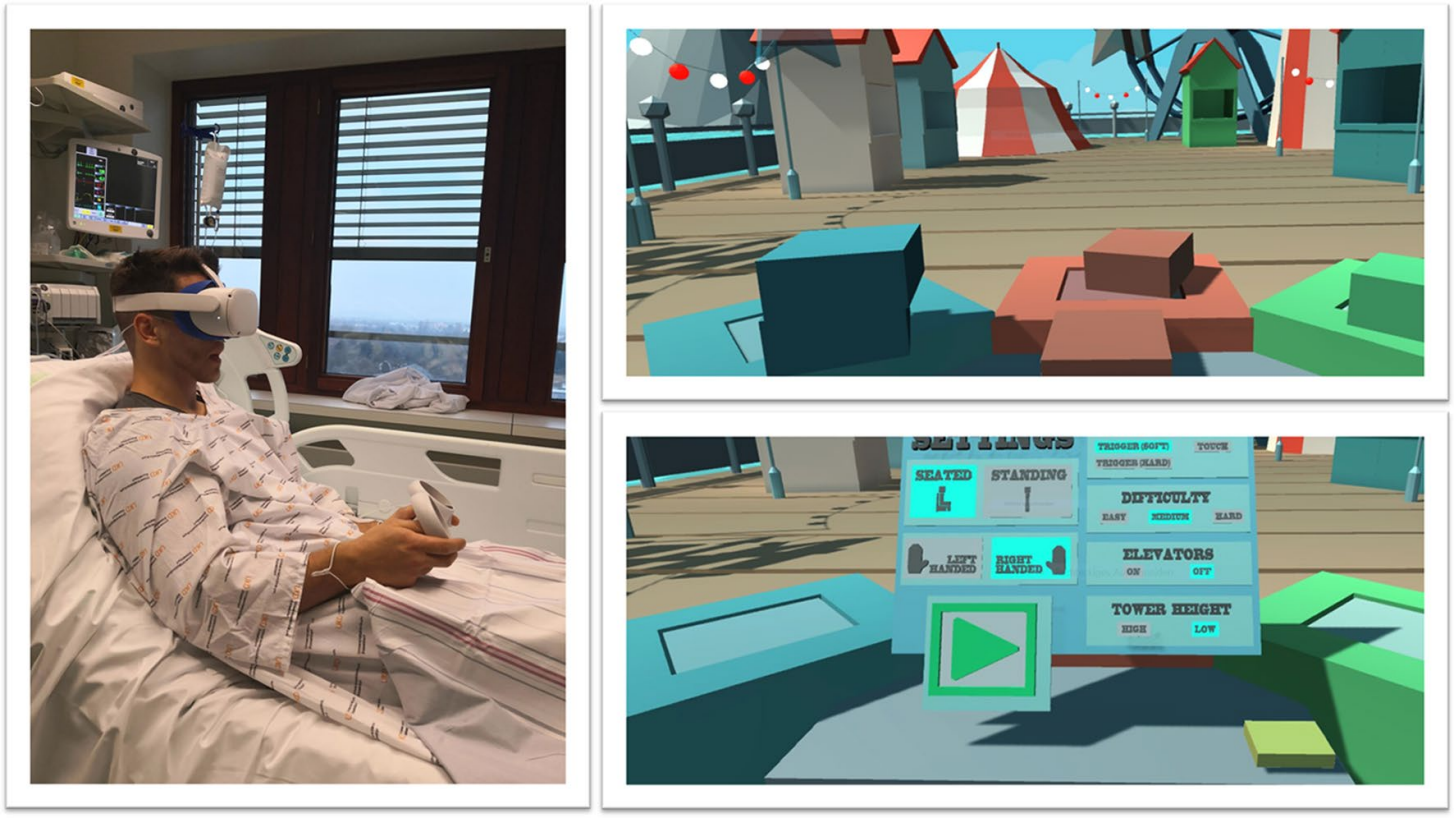
VR with virtual gaming for rehabilitation. With permission from Immersive Rehab Ltd., United Kingdom

“Intensive care unit acquired weakness” during an extended ICU stay is a common phenomenon and has a negative impact on short- and long-term outcomes [25]. VR applications can support rehabilitation programs on the ICU. Gomes et al. integrated a commercially available gaming platform (Nintendo Wii™) into physical therapy sessions in 60 adult ICU patients, with no mobility restrictions, to enhance their physical activity [26]. Activity levels were classified as light to moderate on a modified Borg scale. After 100 sessions, 86% of patients stated that they would like to play the videogame in future physical therapy sessions. The same gaming platform (Nintendo Wii™) was evaluated by Abdulsatar et al. in a pilot-trial with 12 critically ill children [27]. Upper limb activity during Wii™ sessions increased significantly; although grip strength did not change when compared to baseline findings. There were no adverse events attributed to the VR intervention. Although most VR platforms are primarily used in the entertainment industry, specific VR solutions have been designed for health care use. A study conducted by Parke et al. looked to enhance early ICU mobilisation with VR support: 20 adult ICU patients engaged in therapy sessions with the Jintronix virtual therapy system targeting arm, leg, and trunk strength, as well as range of motion, and/or endurance exercises [28]. The primary objective of this investigation, which was achieved, was safety and feasibility. However, almost all participants reported that the VR activity was enjoyable, improved body strength and range of motion, and would motivate them to continue exercising. ImmersiveRehab® is a commercially available VR environment that uses different tasks to enhance rehabilitation after critical illnesses such as stroke (Fig. 5). Additionally, Wang et al. developed a VR application for early mobilisation of critically-ill patients, which has not yet been evaluated in patients or volunteers [29]. In summary, commercially available VR entertainment applications are safe, feasible and well accepted in critically ill patients and might be beneficial in the physical rehabilitation process on the ICU, although randomised-controlled studies are currently lacking.

#### Virtual reality for early neurocognitive stimulation

Up to 60% of ICU survivors suffer from significant long-term neurocognitive impairment [30]. Turon et al. conducted a pilot study on the value of VR-assisted early neurocognitive stimulation in 20 critically ill adult patients undergoing and/or having undergone mechanical ventilation for ≥ 24 h. In brief, the simulation included a virtual avatar that accompanies patients, helps them to orient in time, delivers instructions, motivates them to complete exercises, and encourages them to relax. This VR-assisted neurocognitive intervention was found to be feasible, safe, tolerable, and effectively stimulated cognitive function. However, there was no control group, and no follow-up data were available [31]. To date, there is no evidence from randomised-controlled trials to support the role of VR in reducing neurocognitive impairment, although promising pilot studies exist.

#### Virtual reality after intensive care

Following ICU treatment, many patients suffer from Post Intensive Care Syndrome (PICS), which consists of mental health issues, cognitive dysfunction, and problems with mobility [32]. It was therefore hypothesised that more information on ICU therapy and subsequent medical procedures might be beneficial. Indeed, many ICU patients would like to enhance their knowledge about critical care [33]. Conventional methods, such as written brochures, are either not well accepted or not utilised [33]. A randomised-controlled trial by Vlake et al. aimed to determine whether the repetitive application of VR modules explaining ICU treatment improved subjective well-being and quality of life three and six months after ICU treatment. These modules lasted about 14 min and explained different aspects of ICU treatment that were felt to be the most frightening [34, 35]. In total, 57 ICU patients were randomised to VR, and 47 patients served as a control group. VR resulted in a reduction of post-traumatic stress disorder and lower depression scores. Mental health was better from two days until one month after initial VR exposure. Interestingly, this effect was still present for post-traumatic stress disorder and depression, but not mental quality of life six months after exposure. Regarding safety, cybersickness scores were low, and no changes in vital signs were observed [34, 35]. Recently, the same working group conducted a multicentre randomised-controlled trial including 89 COVID-19 ICU survivors [36]. The VR strategy consisted of a 14-min informational video with different scenes explaining the ICU environment and treatment. The VR intervention was performed during the COVID-19 post-ICU follow-up clinic appointment, three months after hospital discharge. VR did not reduce the psychological distress or quality of life as compared to the control group. However, VR significantly improved subjective satisfaction scores and the overall rating of ICU aftercare. Most VR patients stated that they would recommend ICU-VR to other ICU survivors. In summary, the use of VR after ICU does not improve clinically relevant endpoints, but has a high acceptance rate among patients.

### Virtual reality from the patient relative’s perspective

#### Situational understanding: virtual intensive care unit rounds

Admission to a paediatric intensive care unit poses significant stress and uncertainty on relatives—especially the parents. During the COVID-19 pandemic, parents had limited ability to participate in clinical rounds. As a countermeasure, Tallent et al. developed a VR-based virtual visit to the ICU. The VR-visit did not increase the duration of the ward. [37]. In this study the VR-ICU ward rounds potentially helped to maintain close communication between patients, their relatives, and the health care providers. However, to date, not a single study exists which investigates patient or patient-relative related outcomes in this context.

### Virtual reality from the health care provider’s perspective

#### Virtual Reality for education and training

VR can be used as a tool to train staff how to manage different clinical scenarios and perform clinical skills. [2, 4]. VR has some theoretical advantages compared to “real-life training”: complex activities can be repeated as often as desired, no patients or volunteers are required, no company representative is required for instruction, training can be performed at any given time, and no consumable goods are necessary, which might be associated with significant expenditure. For example, when practicing the priming of extracorporeal membrane oxygenation or other cardiac assist devices, considerable material costs can arise per training session.

Multiple studies have been conducted to test the ability of VR to support learning and training of health care providers. In an ICU setting, Chiang et al. evaluated the success of VR-based learning on tracheostomy care in a prospective, controlled, 2:1 randomised pre–post-study. The interventional group (*n* = 30) received a VR simulation for 15 min, and the control group regular text-based training. VR increased self-efficacy, including familiarity and confidence, and reduced anxiety about tracheostomy-related knowledge and skills compared to the control group. This effect persisted until three to four weeks after the intervention [38]. Yu et al. evaluated the impact of a VR simulation program on Korean nursing students’ knowledge, performance self-efficacy, and learner satisfaction in neonatal critical care [39]. The VR group showed greater improvements in high-risk neonatal infection control performance, self-efficacy and learner satisfaction compared to the control group [39]. Ralston et al. evaluated a VR environment to test the use of VR in simulating paediatric critically ill clinical scenarios. One scenario simulated an ectopic junctional tachycardia and low cardiac output syndrome; the other simulated an acute respiratory failure in a patient with suspected Covid-19 infection [40]. Although there was no control group, all six paediatric cardiac critical care physicians successfully navigated the VR environment.

**Fig. 6:**
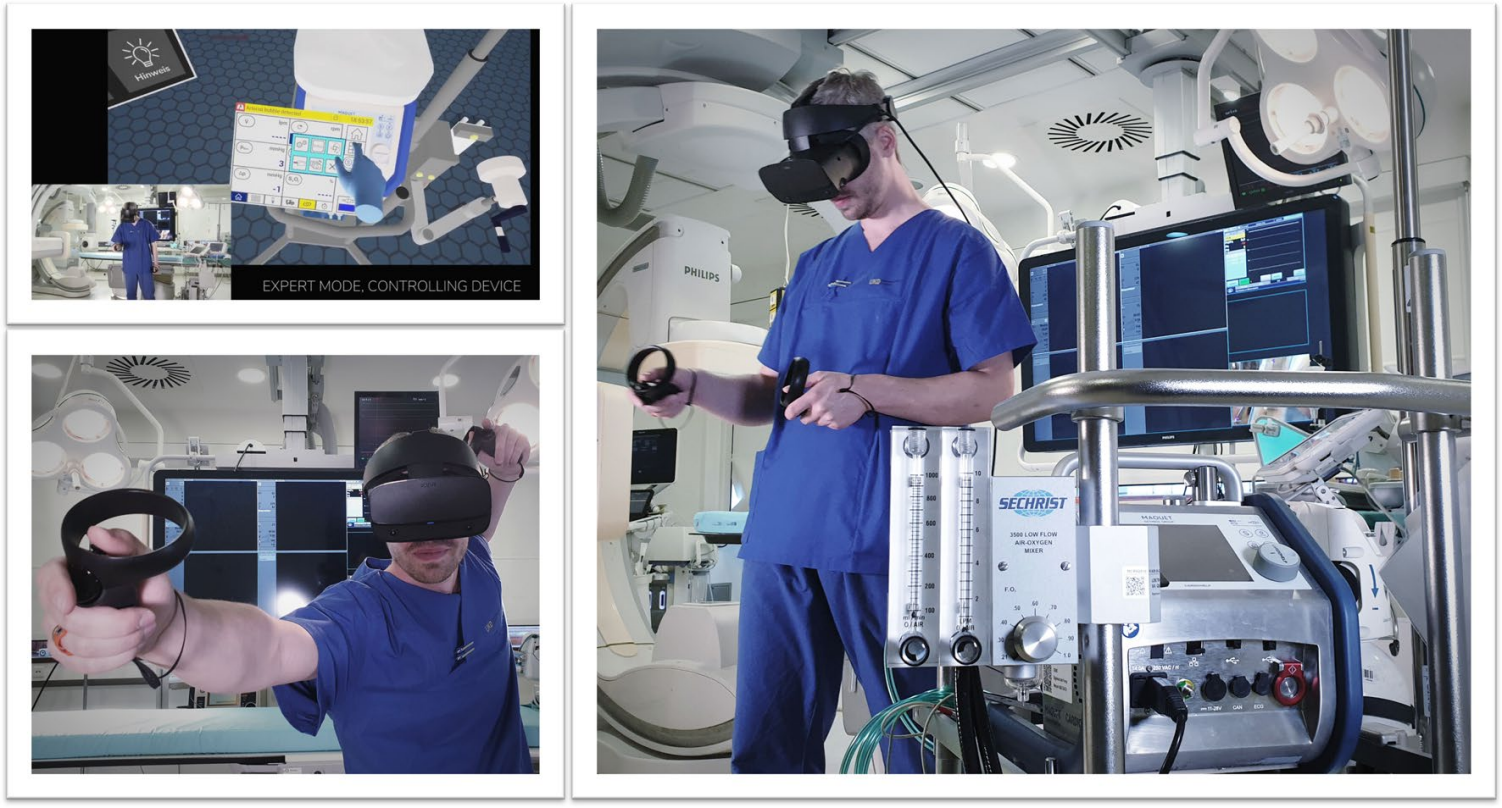
VR for health care providers to train in complex procedures. With permission from Weltenmacher®, Germany

Agasthya et al. evaluated the value of a 19-min immersive tutorial (interventional group) on intubating an infant manikin, in a controlled trial. The primary endpoint (the performance accuracy measured by a checklist) did not differ between groups [41]. Over 20 years ago, Colt et al. established a VR bronchoscopy simulation for critical care medicine. After VR-training, five novice physicians had comparable skills, in terms of dexterity, speed, and accuracy, to four experienced physicians [42]. Farra et al. compared the success of VR emergency evacuation training versus web-based clinical updates in a neonatal critical care unit. Both approaches did not statistically differ in their perceived self-efficacy, although the VR group performed statistically better in the live exercise [43]. Recently, Wolff et al. developed a VR environment consisting of different steps in ECMO-priming (Fig. 6) [4]. In summary, VR or AR might be a complementary, but not a substitution, for training health care providers in basic and advanced life support. In this context, currently available data show heterogeneous results [44].

#### Virtual reality for stress relief

Stress affects ICU health care providers, potentially resulting in burnout and decreased productivity [45]. Nijland et al. evaluated the impact of VR on the self-perceived stress level of 66 ICU nurses during their breaks. Sixty-two percent of those stated that VR was helpful in reducing stress [46]. Gerber et al. evaluated the stress relieving effect of VR in 45 healthy subjects: dynamic, virtual, natural, and urban environments were presented inside the head-mounted display and a neutral video on an ICU television screen. The natural environment had the highest positive and restorative impact on the subject’s physiological and psychological state [47]. Furthermore, ICU caregivers enjoyed pleasant artificial VR environments during their breaks [48].

#### Augmented reality for training

AR can assist health care providers in critical care procedures, such as intubation or central line placement. Alismail et al. conducted a controlled trial with 32 ICU trainees. The AR group (15 participants) used head-mounted AR glasses during endotracheal intubation of a training doll. The AR display repeated the essential, practical steps. The interventional group needed more time to intubate and ventilate, but had a higher adherence to evidence-based intubation practice [49]. Airway management is of pivotal importance in neonatal ICUs [50]. Dias et al. compared three learning strategies for endotracheal intubation in ICU nurses: direct laryngoscopy, indirect video laryngoscopy and AR-assisted video laryngoscopy with a magnified video of the airway alongside normal vision. AR-assisted video laryngoscopy was not inferior to normal indirect video laryngoscopy and safer than direct laryngoscopy. Huang et al. used a similar AR-based approach for the training of central venous line placement. Although, there was no difference in procedure time, there was a higher adherence to the procedure check list in the AR group (*p* = 0.003) [51]. Heo et al. conducted a prospective, controlled pilot study, randomising nurses with no prior experience in mechanical ventilation to conventional training or AR-assisted training. In the AR-group, the nurses were guided by AR-based instructions and could request assistance using the head-mounted display. AR resulted in a lower need for assistance compared to the manual group and a higher level of confidence after training [52].

AR can also be used to assess the mental and physical status of patients more accurately and may improve the recognition of deteriorating vital signs. In a trial by Zackoff et al., ICU teams completed two critical care scenarios: first, traditional training using a manikin, then AR-enhanced training using a manikin. AR improved the ability to assess the patient's mental status, respiratory status, and perfusion status, as well as recognition of hypoxemia, shock, apnoea and decompensation, but not the recognition of cardiac arrest.

#### Augmented reality in performing invasive procedures

Central line placement and endotracheal intubation are standard ICU procedures but can be associated with severe complications. Percutaneous dilatational tracheostomy is a frequently performed intervention on the ICU. In this context, Gan et al. used AR in six patients undergoing the aforementioned procedure with “good success and excellent user feedback” [53]. The use of an AR-assisted near-infrared electromagnetic radiation device in older ICU patients undergoing venous puncture lowered the incidence of hematomas in venous puncture but did not decrease procedure length or the number of attempts [54]. Yamada et al. developed an AR interface for smartphones and tablets that can be used by ECMO-perfusionists [55]. However, to date there are no studies evaluating its effectiveness compared to traditional learning methods. Similarly, Scquizzato et al. proposed an AR based smartphone application for estimating the weight of critically ill paediatric patients, but it has not been evaluated in a clinical setting.[56]. In conclusion, there is currently no convincing evidence for or against the use of AR-supported invasive procedures in critical care medicine.

## AR/VR from a clinician’s perspective

There are a number of promising indications for AR/VR use in critical care medicine, which could be integrated into daily practice. VR could be part of a multimodal strategy, used to reduce analgesic requirements. Likewise, VR may help to support cognitive stimulation and physical activity. However, AR/VR applications are not designed to, and will not be able to, replace personal communication. Patients and their relatives welcome VR-assisted information about ICU procedures [57]. A similar conclusion applies to VR-based training for health care providers: there are promising approaches to support, but not to replace, traditional learning techniques. To date, there is no convincing evidence for the role of AR-supported practical procedures, such as endotracheal intubation or central venous line placement in critical care medicine outside of clinical trials.

### The “vergence accommodation conflict”, cybersickness and possible solutions

VR can cause side effects such as headache, nausea and vomiting—so-called “cybersickness”—which can be related to motion sickness [58]. Cybersickness is not yet a defined health condition. Motion sickness occurs due to a difference between actual and expected motion. However, this pathophysiological mechanism may not be 100% transferrable to cybersickness. The “vergence accommodation conflict” during VR sessions also plays a role. This phenomenon arises because wearing the VR glasses leads to a disparity between the physical surface of the screen (“accommodation”) and the focal point of the virtual simulated world the user gazes at (“vergence”). This disparity can lead to nausea, headache, and discomfort. At the moment, several possible solutions to the “vergence accommodation conflict” are under evaluation [59], which potentially challenges the broad application of VR in medical training [60]. However, cybersickness might be stronger in AR than VR: in one study, 15.3% of participant reported headache and 17 other symptoms, including nausea, after using AR-based training for gross anatomy dissection (HoloAnatomy®) [61]. By contrast, Bruno et al. found no increased signs of cybersickness during their pilot study, which used VR to distract patients during transcatheter aortic valve implantation [6]. AR/VR related side-effects seem to vary among different age and gender groups [62]; an effect which is not yet fully understood and needs further investigation. Thus, the cornerstone of VR-based application might be careful patient selection and prompt assistance should side effects occur.

## VR/AR from an ethical perspective

In vulnerable patient groups, such as critically ill patients, there are some ethical concerns regarding the use of VR/AR. For this purpose, Kellermeyer et al. established three core principles [63]:

1. If there is a choice, a human-to-human interaction should be preferred (“therapeutic alternativism”) over human-to-machine interaction (no “technological solutionism”).
2. VR technology should centre around “critical human values,” including dignity and autonomy (“human-oriented value alignment”).
3. VR systems should be patient centred, not focusing on the need of professional customers (“patient-centered design”).

From our point of view, these principles are of pivotal importance. VR/AR should always enhance the real-world provider—patient-relationship and should not be a tool to replace it. Some researchers proposed the creation of a new medical specialty, the “virtualist”, who undergoes extensive technical and medical training, but also has a deep understanding of the ethical implications of VR/AR technologies [64]. We believe that critical care physicians and patient representatives should actively participate in the development and continuous improvement of all virtual and digital technologies to ensure they are user-friendly and patient-centred.

## VR/AR from a researcher’s perspective

There are a number of difficulties surrounding clinical studies using VR/AR applications. Namely, due to the extensive range of VR/AR glasses (hardware) and software it is extremely difficult to make a direct comparison. In fact, both components are often tested simultaneously in one trial, which may lead to interaction and a lack of clarity in the interpretation of results. [65]. In future studies, protocols and endpoint definitions should be harmonised as much as possible. The software used differs considerably. Some studies simply use commercially available devices and software (e.g. Nintendo Wii [27]) while others—such as physicians and researchers—customise the software from existing VR environments to specific patient/educational needs [6]. Additionally, some manufacturers specifically produce the exact software to create the environment required for the clinical purpose (ImmersiveRehab ^Ltd^ or Healthy Mind®). Most studies are "proof-of-principle" approaches focusing on the feasibility and safety of a specific VR/AR application.

Another problem is that VR hardware is rapidly evolving: head-mounted displays are generating ever-higher graphics resolution, easier interactivity, and, thus, greater immersion. Therefore, studies using the latest VR hardware demonstrate greater utility than older devices.

Unfortunately, the degree of immersion and occurrence of cybersickness are rarely measured or reported, although the effect of VR crucially depends on it [66]. Complex scores have been developed and validated for this purpose. The Simulator Sickness Questionnaire (SSQ), for example, uses 16 questions with four levels of severity to examine "nausea, oculomotor problems and disorientation" [67].

Currently, there is a lack of prospectively randomised controlled trials in this area of research. In addition, none of the studies were blinded. Theoretically, the intervention group could be compared with a control group, in which “sham VR applications” are used. “Sham VR applications” could consist of using VR glasses with no specific digital content. It is often difficult to distinguish between the relative benefits of immersive VR compared with established non-pharmacological distraction methods such as relaxation techniques or music therapy. At a minimum, investigators should be blinded to reduce bias.

In summary, future studies should consider the following aspects:

1. Methodical separation of software and hardware.
2. A detailed statement of the software development and validation process.
3. Prospective trial design with a randomised-controlled recruitment.
4. If possible, double blinding, but at least single blinding should be ensured.
5. Degree of immersion measurement.
6. Structured recording of "cybersickness" using validated scores.
7. Descriptive measures of “usual care” in the control group.

## Conclusion and future directions

With the ongoing COVID-19 pandemic, innovative VR and AR applications offer new solutions for many aspects of daily critical care medicine. With advancing data transfer speeds; additional applications are emerging, such as remote distance treatment and care. Currently, remote treatments using robotic devices are under development [68]. This might enable independent, high-quality care in remote locations where expertise is unavailable. We believe that VR and AR will soon become mainstream reality in ICUs all over the globe. To create evidence-based knowledge, particular attention should be paid to consistent research design in further (clinical) trials.

## Data Availability

We did not use and individual participant or patient data.

## Abbreviations

3D: Three dimensional
AR: Augmented reality
CPR: Cardiopulmonary resuscitation
CT: Computed tomography scan
ECMO: Extracorporeal membrane oxygenation
ICU: Intensive care unit
TAVR: Transcatheter aortic valve replacement
PTSD: Post-traumatic stress disorder
VR: Virtual reality
